# Integrated Genomic and Functional Characterization of *Lactiplantibacillus plantarum* MS11 Reveals Multifunctional Metabolite Production from a High-Altitude Fermented Dairy Niche

**DOI:** 10.3390/microorganisms14040854

**Published:** 2026-04-10

**Authors:** Yixuan Lin, Qi Liang, Baotang Zhao, Xuhui Chen, Xuemei Song

**Affiliations:** Functional Dairy Products Engineering Laboratory of Gansu Province, College of Food Science and Engineering, Gansu Agricultural University, Lanzhou 730070, China

**Keywords:** *Lactiplantibacillus plantarum*, whole-genome sequencing, folate, L-lactic acid, bacteriocin, EPS

## Abstract

*Lactiplantibacillus plantarum* MS11, isolated from traditionally fermented yak milk in the high-altitude Gannan region of the eastern Tibetan Plateau, was investigated for its technological and functional potential in food applications. Using whole-genome sequencing combined with targeted experimental verification, this study clarified the genetic determinants and metabolic capacity associated with its production of folate, lactic acid, bacteriocin, and exopolysaccharides (EPS). The MS11 genome consists of one circular chromosome and three plasmids, totaling 3,318,231 bp with a GC content of 44.48%, and encodes 3155 predicted open reading frames. Complete biosynthetic gene clusters were identified for folate (7 genes), L-lactic acid (13 genes), bacteriocin (14 genes), and EPS (17 genes). Phenotypic assays confirmed the strain’s high metabolite productivity, including folate (0.6043 μg/mL), L-lactic acid (76.24 mg/mL), and EPS (544.2 mg/L). The cell-free fermented supernatant exhibited strong antibacterial activity against *Escherichia coli*, supporting the functional relevance of its bacteriocin-associated gene cluster. To the best of our knowledge, this is the integrated genomic and experimental characterization demonstrating that a *L. plantarum* strain originating from a unique high-altitude fermented dairy niche can concurrently synthesize high levels of folate together with multiple beneficial metabolites. The multifunctional attributes of MS11—including nutrient fortification, acidification capacity, EPS formation, and antimicrobial activity—indicate substantial promise for its application as a composite starter culture, natural bio-preservative, and nutritionally enhanced probiotic in fermented food systems.

## 1. Introduction

Probiotics have garnered considerable interest in the life sciences due to their demonstrated roles in promoting gut health, modulating immunity, and aiding disease management [1,2,3]. Among these, *Lactiplantibacillus plantarum* is included in authoritative lists of strains permitted for use in foods [4] and is widely employed in food fermentation and probiotic product development [5,6,7]. The functional and metabolic diversity of *L. plantarum* is closely linked to its ecological source [8,9]. Metabolites produced by *L. plantarum* contribute to its probiotic effects. The lactic acid it synthesizes can lower intestinal pH, inhibit pathogens, and help regulate gut microecological balance [10,11,12]. Certain strains also produce vitamins such as folate, which plays a role in preventing anemia and Alzheimer’s disease [13]. Additionally, some strains synthesize bacteriocins—low-molecular-weight, cationic, heat-stable antimicrobial peptides active against Gram-positive and Gram-negative bacteria [14,15,16]. These peptides are regarded as natural bio-preservatives for controlling spoilage and pathogenic microorganisms in foods, and they also exhibit immunomodulatory potential [3,17,18,19]. Exopolysaccharides (EPS) are another key functional metabolite. These structurally complex carbohydrates are natural, biodegradable, and safe macromolecules with demonstrated bioactivities, including antitumor, antioxidant, and immunomodulatory effects, granting them significant value in the food and biomedical sectors [1,20,21,22]. Therefore, the discovery and characterization of novel *L. plantarum* strains capable of coordinately producing multiple high-value metabolites—such as organic acids, vitamins, bacteriocins, and EPS—holds considerable importance for developing advanced starter cultures and multifunctional probiotic products.

While traditional phenotypic methods offer insights into some metabolic traits of *L. plantarum*, their time-consuming and low-throughput nature limits systematic exploration of its full metabolic potential [23]. Furthermore, research has often focused on evaluating or optimizing single-strain functions [24], with insufficient systematic analysis of isolates from unique ecological niches that may harbor distinctive, synergistic metabolic capabilities. This gap is particularly evident in studies that comprehensively link genomic potential to validated phenotypic outputs. Although the genomes and probiotic functions of several *L. plantarum* strains are well documented [25,26,27], most efforts have centered on model or commercial isolates. Extreme environments, such as the high-altitude eastern fringe of the Tibetan Plateau—characterized by an average elevation >3000 m, a mean annual temperature of 1.7 °C, and a cold, humid climate [28]—constitute a valuable reservoir for screening strains with unique functional attributes. Genomes shaped by such niches may integrate adaptive genetic modules that support survival under extreme conditions while enabling the efficient, coordinated synthesis of multiple functional metabolites. Currently, there remains a significant lack of research that elucidates the genetic basis and provides experimental validation for such efficient metabolism in strains derived from these distinctive environments.

This study focuses on the strain *L. plantarum* MS11, isolated from traditionally fermented yak milk in the high-altitude region of the Gannan Tibetan Autonomous Prefecture on the eastern Tibetan Plateau. This strain exhibits the synergistic coordination of four major functional characteristics: efficient folate synthesis, high-yield production of L-lactic acid, bacteriocin secretion, and high-yield production of exopolysaccharides (EPS). Its performance in each of these areas meets or exceeds that of strains specialized in a single function, thereby overcoming the limitations of traditional strains, which typically possess a single function and are difficult to optimize across multiple traits. Furthermore, originating from an extreme environment characterized by high altitude, low temperature, and low oxygen, the genome of MS11 integrates unique environmental adaptation modules, making it significantly superior to strains from conventional sources in low-temperature fermentation and enabling stable maintenance of multi-functional metabolic activity under extreme conditions. Consequently, MS11 combines three key application attributes—nutritional fortification, biopreservation, and probiotic functionality—enabling the one-step development of composite microbial agents. This strain, therefore, offers a unique composite application value that cannot be achieved by existing single-function strains.

## 2. Materials and Methods

### 2.1. Materials

*L. plantarum* MS11 was previously isolated from fermented yak milk in Hezuo City, Gannan Tibetan Autonomous Prefecture, eastern Tibetan Plateau, and preserved by the Gansu Provincial Functional Dairy Engineering Laboratory.

The *Escherichia coli* strain used in this study was obtained from the Microbial Culture Collection of the College of Food Science and Engineering, Gansu Agricultural University.

### 2.2. Methods

#### 2.2.1. Isolation of Genomic DNA

*L. plantarum* MS11 was revived and cultured using the following procedure: First, the cryopreserved stock (−80 °C) was streaked onto agar plates for isolation. A well-isolated single colony was then inoculated into de Man, Rogosa, and Sharpe (MRS) broth and incubated at 37 °C for 12 h. Subsequently, bacterial cells were harvested by centrifugation and submitted to Personal Biotechnology Co., Ltd. (Shanghai, China) for genomic DNA extraction, sequencing, and library construction.

Genomic DNA was isolated from MS11 using the cetyltrimethylammonium bromide (CTAB) method. The concentration and purity of the extracted DNA were then assessed using a Qubit Fluorometer (Thermo Fisher Scientific, Carlsbad, CA, USA) and a NanoDrop spectrophotometer (Thermo Fisher Scientific, Wilmington, DE, USA). Furthermore, DNA integrity and the absence of significant RNA or protein contamination were verified by electrophoresis on a 1% (*w*/*v*) agarose gel.

#### 2.2.2. Library Preparation and Sequencing

Genomic libraries were prepared using Illumina TruSeq (Illumina, Inc., San Diego, CA, USA) and Oxford Nanopore kits (Oxford Nanopore Technologies plc, Oxford, UK), followed by whole-genome sequencing on the NovaSeq 6000 (Illumina, Inc., San Diego, CA, USA) and PromethION 48 (Oxford Nanopore Technologies plc, Oxford, UK) platforms, respectively. Raw reads were quality-controlled with AdapterRemoval and SOAPec. De novo assembly was performed separately using SPAdes (v4.2.0) (Illumina data) and A5-miseq (http://sourceforge.net/projects/ngopt, 25 August 2025) (Nanopore data). A hybrid assembly integrating both datasets was then generated with Flye (2.9.6) and Unicycler (v0.5.1), and iteratively polished using Pilon (1.24) to yield a high-quality, complete genome. Finally, open reading frames (ORFs) were predicted with GeneMarkS (v.4.30), and a circular genome map was generated using CGView (1.6.0).

#### 2.2.3. Annotation of Protein-Coding Genes and Identification of Probiotic-Associated Genes

A functional annotation pipeline was applied to the coding sequences of *L. plantarum* MS11 using multiple databases. Gene Ontology (GO) terms were assigned across biological processes, molecular functions, and cellular components. Metabolic pathways and signal transduction networks were annotated using the Kyoto Encyclopedia of Genes and Genomes (KEGG). Evolutionary and orthologous relationships were analyzed by integrating the eggNOG and NR databases to identify conserved and species-specific functions. All annotations were cross-validated to ensure accuracy and consistency.

#### 2.2.4. Biosafety Evaluation of *L. plantarum* MS11

To identify potential antimicrobial resistance (AMR) genes, the annotated genome of *L. plantarum* MS11 was screened against the Comprehensive Antibiotic Resistance Database (CARD) and the ResFinder platform (4.7.2). Similarly, virulence-associated genes were identified using the Virulence Factors Database (VFDB).

#### 2.2.5. HPLC Analysis of Folate

Folate concentration was quantified according to the method described by Hosseini et al. [29], with minor modifications. During the culturing of the third-generation culture of *L. plantarum* MS11, the folate content in the bacterial suspension was measured at 2 h intervals using high-performance liquid chromatography (HPLC). Three biological replicates were prepared for each time point, and a time-course curve of folate content was constructed based on these replicates.

(1) HPLC Conditions

Chromatographic separation was performed on a C18 column. Mobile phase A comprised 100% PBS solution (500 mL), and mobile phase B comprised acetonitrile solution (500 mL). Both solvents were filtered through a 0.22 μm membrane filter and then degassed via ultrasonication for 15 min. The flow rate was set to 1 mL/min, the column temperature was maintained at 30 °C, the detection wavelength was 254 nm, the injection volume was 10 μL, and the retention time was 1.611 min.

(2) Calibration Curve Establishment and Sample Preparation

A folate standard stock solution (1000 μg/mL) was prepared by dissolving 10.0 mg of folate in 0.1 mol/L NaOH containing 0.1% (*w*/*v*) ascorbic acid, diluted to 10 mL. Working standards (0.25–2.0 μg/mL) were prepared by serial dilution with the same solvent. For samples, bacterial cultures were ultrasonicated for 10 min, centrifuged at 12,000× *g* for 5 min, and the supernatant was collected. All standards and supernatants were filtered through a 0.22-μm membrane prior to HPLC analysis within 24 h. A calibration curve was generated by plotting chromatographic peak area against folate concentration.

#### 2.2.6. HPLC Analysis of L-Lactic Acid

Fermentation parameters were assessed according to the method described by Anh Ngoc Le et al. [30], with minor modifications. During the culturing of the third-generation culture of *L. plantarum* MS11, the pH, OD value, and L-lactic acid content of the bacterial suspension were measured at 2 h intervals using a pH meter (PHS-25, Shanghai INESA Scientific Instrument Co., Ltd., Shanghai, China), a microplate reader (RT-6000, Shenzhen Rayto Life Science Co., Ltd., Shenzhen, China), and a high-performance liquid chromatography system (LC-16, Shimadzu Instruments Co., Ltd., Suzhou, China), respectively. Three biological replicates were prepared for each time point, and time-course curves for pH, OD value, and L-lactic acid content were constructed based on these replicates.

(1) HPLC Conditions

Chromatographic separation was performed on a C18 column. Mobile phase A comprised 5 mmol/L H_2_SO_4_ solution (500 mL), and mobile phase B comprised acetonitrile solution (500 mL). Both solvents were filtered through a 0.22 μm membrane filter and then degassed via ultrasonication for 15 min. The flow rate was set to 0.6 mL/min, the column temperature was maintained at 35 °C, the detection wavelength was 210 nm, the injection volume was 10 μL, and the retention time was 6.111 min.

(2) Calibration Curve Establishment and Sample Preparation

An L-lactate stock solution (10 mg/mL) was prepared by dissolving 100 mg of sodium L-lactate in ultrapure water, diluted to 10 mL. Working standards (0.05–5.0 mg/mL) were obtained by serial dilution. For sample preparation, bacterial cultures were acidified with 10 mmol/L H_2_SO_4_ to 1% (*v*/*v*), incubated on ice for 10 min, and centrifuged at 12,000× *g* for 15 min. The supernatant was mixed with an equal volume of acetonitrile, incubated on ice for 15 min, and centrifuged again to remove proteins. The resulting supernatant was diluted 10- to 100-fold with 5 mmol/L H_2_SO_4_ to fit the calibration range. All standards and processed samples were filtered through a 0.22-μm membrane and analyzed by HPLC within 24 h. A calibration curve was plotted using chromatographic peak area versus L-lactate concentration.

#### 2.2.7. Ammonium Sulfate Precipitation

The extraction of bacteriocin-like substances was performed according to the method of Gu et al. [31], with minor modifications. Ammonium sulfate precipitation was performed. The cell-free fermentation supernatant of *L. plantarum* MS11 was adjusted to pH 7 with NaOH, and catalase was then added. The mixture was centrifuged at 10,000× *g* for 10 min, and the supernatant (200 mL) was collected. Ammonium sulfate powder was slowly added to reach a final saturation of 70%, and the mixture was gently shaken until the salt was completely dissolved. After incubation at 4 °C for 24 h, the mixture was centrifuged at 10,000× *g* for 10 min, and the supernatant was discarded to obtain a brownish precipitate, which constituted the crude bacteriocin extract. Bacteriocin activity was assessed using the double-layer agar plate diffusion method. The diameter of the inhibition zone produced by the crude bacteriocin extract against *E. coli* was measured using a Vernier caliper, and readings were recorded to an accuracy of 0.01 mm.

#### 2.2.8. Phenol-Sulfate Assay

This assay was performed according to the method described by Zhao et al. [32], with minor modifications.

(1) Calibration Curve Establishment

After drying to constant weight, 100.0000 mg of glucose standard was accurately weighed, dissolved in 80 mL of ultrapure water, and diluted to a final volume of 100 mL. A 5 mL aliquot of this solution was transferred into a 50 mL volumetric flask and diluted to volume with ultrapure water to obtain a glucose standard solution (0.1 mg/mL). Aliquots of 0, 0.2, 0.4, 0.6, 0.8, and 1.0 mL of the glucose standard solution were added into separate test tubes, and the volume was adjusted to 1 mL with ultrapure water. Subsequently, 1 mL of 6% phenol solution and 5 mL of concentrated H_2_SO_4_ were added sequentially to each tube. After allowing the samples to stand until they reached room temperature, the absorbance at 490 nm (A_490_) was measured using a UV spectrophotometer (UV-1280, Shimadzu Instruments Co., Ltd., Suzhou, China). A standard curve was plotted with the concentration of the glucose standard solution as the abscissa and the absorbance as the ordinate.

(2) Sample Preparation

After the culturing of *L. plantarum* MS11 was completed, the culture broth was centrifuged at 8000× *g* and 4 °C for 10 min. The supernatant was collected and sterilized by filtration using a 0.22 μm membrane to completely remove residual bacterial cells, thereby yielding the cell-free fermentation supernatant. Three volumes of ethanol were added to the cell-free fermentation supernatant, and the mixture was incubated at 4 °C for 24 h, followed by centrifugation at 12,000× *g* for 30 min to collect the precipitate. Trichloroacetic acid (80%, *w*/*v*) was added to the solution to achieve a final concentration of 4% for protein removal. After incubation at 4 °C for 12 h, the mixture was centrifuged at 12,000× *g* for 15 min. Following phase separation, the intermediate denatured protein layer and the lower organic phase were discarded to thoroughly remove protein impurities from the sample. The deproteinized EPS solution was transferred into a dialysis bag with a molecular weight cutoff (MWCO) of 8000–14,000 Da and dialyzed against a large volume of sterile deionized water at 4 °C for 72 h, during which the deionized water was replaced every 6–8 h to ensure complete removal of small-molecule impurities such as monosaccharides, oligosaccharides, and inorganic salts. After dialysis, the EPS solution inside the bag was collected and freeze-dried to obtain purified EPS powder, which was stored at –20 °C in the dark for subsequent use. The total sugar content of the purified EPS was quantified using the phenol-sulfuric acid method, with glucose as the standard to generate a standard curve. Three biological replicates were prepared for the samples, and the average value was defined as the EPS yield of the strain.

## 3. Results

### 3.1. Genomic Characteristics of L. plantarum MS11

**Figure 1:**
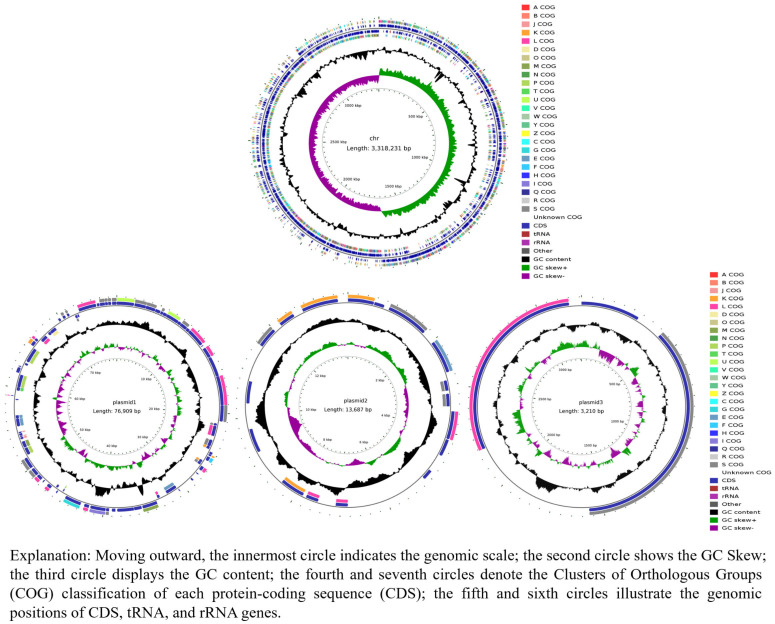
Circular genome map of *L. plantarum* MS11.

Whole-genome sequencing revealed a total genome length for *L. plantarum* MS11 of 3,318,231 bp and a GC content of 44.48%. The genome assembly revealed three plasmids with lengths of 76,909 bp, 13,687 bp, and 3210 bp, and corresponding GC contents of 37.94%, 35.73%, and 38.72%, respectively (Table 1). Gene annotation identified 3155 open reading frames (ORFs), with a cumulative length of 2,773,647 bp and an average GC content of 45.58%, which constitutes 83.59% of the total genome. The genome encodes six 5S rRNA, five 16S rRNA, and five 23S rRNA genes, which collectively form complete ribosomal RNA operons. Additionally, 68 tRNA and 56 ncRNA genes were predicted, indicating a comprehensive network for genetic information transfer and regulation. These genomic features—including the functional distribution of genes based on COG classification, GC content variation, genomic island locations, and a comparative analysis of homologous gene clusters—are summarized in the genome circle map (Figure 1).

### 3.2. The Phylogenetic Tree of L. plantarum MS11 Was Constructed

**Figure 2:**
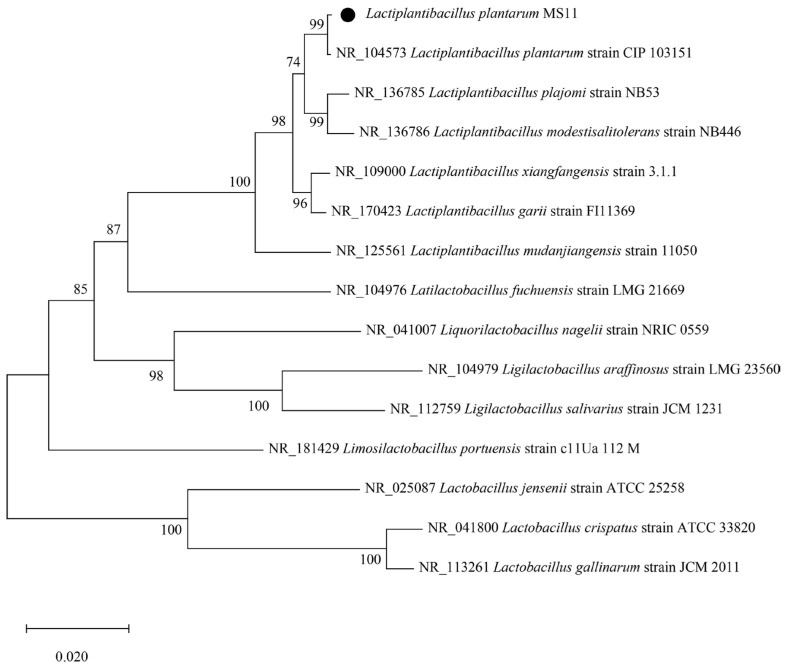
Phylogenetic tree of *L. plantarum* MS11.

Based on 16S rDNA sequencing and phylogenetic tree analysis, strain MS11 was identified as belonging to *Lactiplantibacillus plantarum*. Phylogenetic analysis revealed that strain MS11 is most closely related to *Lactiplantibacillus plantarum* strain CIP 103151 (Figure 2). Furthermore, strain MS11 was found to possess gene clusters homologous to those identified in the reference strain CIP 103151. Specifically, these gene clusters contain key functional genes associated with probiotic properties, notably those encoding bile salt hydrolase (BSH), acid tolerance-related proteins, adhesion factors, folate, lactic acid, bacteriocins, and exopolysaccharides. The presence of these genes confirms the potential of strain MS11 to survive in the gastrointestinal tract, adhere to the intestinal mucosa, and exert antagonistic effects against pathogenic bacteria, constituting core characteristics of probiotic strains.

### 3.3. Genomic Functional Annotation of L. plantarum MS11

To elucidate the functional landscape of *L. plantarum* MS11, its predicted coding sequences were systematically annotated against multiple databases (Table 2). Evolutionary relationships and putative orthologs were assigned using the Non-Redundant (NR) and eggNOG databases. Metabolic pathways and cellular signaling networks were mapped using the Kyoto Encyclopedia of Genes and Genomes (KEGG). Functional categorization was performed with the Gene Ontology (GO) database, assigning genes to the hierarchical categories of biological process, molecular function, and cellular component. High-confidence annotations were curated with the manually reviewed Swiss-Prot database. Protein domain architecture and transmembrane transport functions were characterized using the Pfam and Transporter Classification Database (TCDB), respectively. To resolve conflicting annotations, a hierarchical consensus approach was implemented. Annotations were prioritized based on the highest sequence similarity and query coverage, ensuring reliable final assignments.

#### 3.3.1. KEGG Pathway Analysis

**Figure 3:**
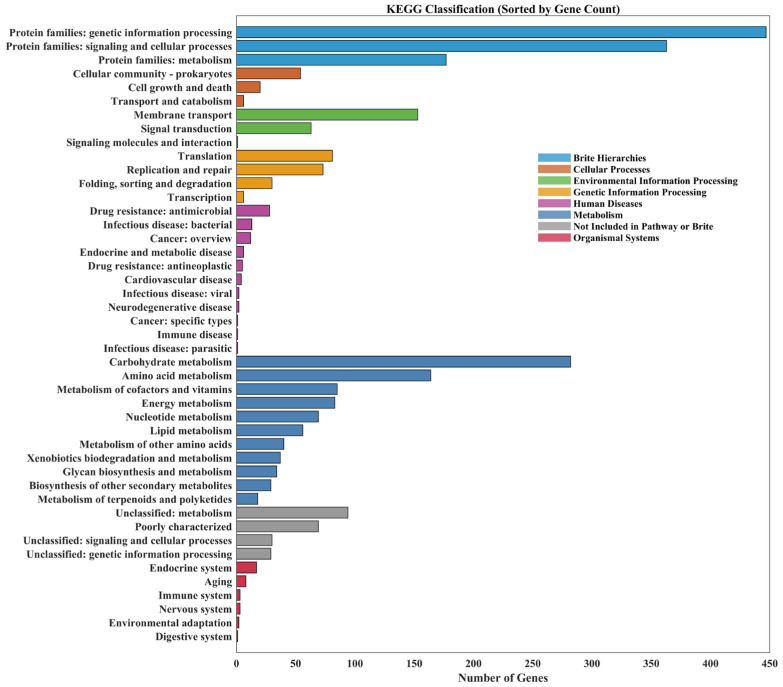
KEGG analysis.

KEGG-based functional annotation of the *L. plantarum* MS11 genome assigned roles to 1424 genes, revealing significant enrichment in metabolic and genetic information processing pathways (Figure 3). This highlights a strong genomic basis for active metabolism and regulation. Specifically, 897 genes are involved in core metabolic processes (carbohydrate, lipid, and amino acid metabolism), indicating efficient nutrient utilization and adaptation to fermentation environments. Environmental information processing includes 217 genes, primarily for membrane transport (153) and signal transduction (63), supporting environmental sensing and response. Genetic information processing encompasses 189 genes related to translation, replication/repair, protein folding/degradation, and transcription, ensuring genetic stability and protein synthesis. Additionally, 80 genes are linked to cellular processes such as biofilm formation, and 75 genes are associated with human disease pathways, offering genetic insight into potential probiotic mechanisms. Overall, this analysis elucidates the metabolic diversity and adaptive strategies of MS11, predicting its potential for industrial fermentation and probiotic applications.

#### 3.3.2. GO Analysis

**Figure 4:**
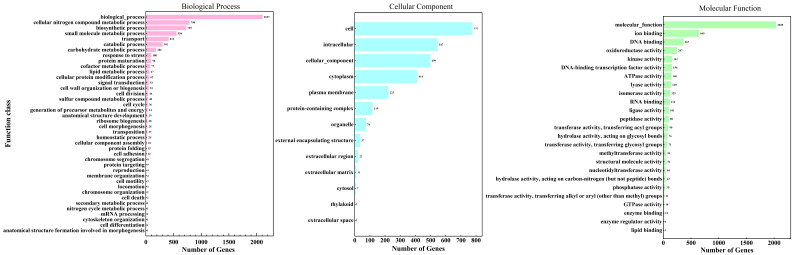
GO analysis.

A genome-wide functional annotation of *L. plantarum* MS11 was performed using the Gene Ontology (GO) database, revealing the distribution of its genes across the three primary GO domains: cellular component, molecular function, and biological process (Figure 4). A total of 2220 genes were assigned GO terms. Among these, 65.20% were assigned to biological processes, reflecting the genetic foundation for dynamic cellular activities such as metabolic regulation, stress response, and cell cycle control. Furthermore, 24.10% were linked to cellular components, predominantly those constituting structures such as the cell membrane and cell wall, which are fundamental to environmental adaptability and cellular integrity. The remaining 6.90% were implicated in molecular functions, encompassing key biochemical activities such as catalysis, binding, and transport. Collectively, these annotations delineate the metabolic network and cellular architecture of MS11 from a functional genomics perspective, thereby establishing a bioinformatic framework for elucidating its potential probiotic mechanisms, including fermentation adaptability, stress tolerance, and host-microbe interactions.

#### 3.3.3. eggNOG Analysis

**Figure 5:**
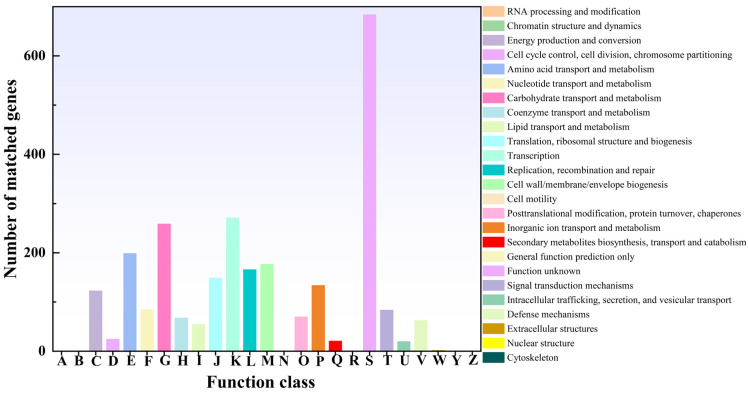
eggNOG analysis.

Building on evolutionary lineage and orthologous protein cluster analysis via the eggNOG database, a systematic COG/KOG functional annotation was conducted for *L. plantarum* MS11. In total, 2655 coding sequences (CDSs) were classified into 19 major COG categories (Figure 5), encompassing functions such as carbohydrate transport and metabolism, amino acid transport and metabolism, energy production and conversion, transcription, translation, replication, recombination and repair, and defense mechanisms. Notably, MS11 showed significant enrichment of genes within carbohydrate metabolism-related categories, corroborating its capacity for efficient utilization of diverse carbon sources. Concurrently, a substantial genetic repertoire was identified in functional groups linked to environmental adaptation and stress response, including those for secondary metabolite biosynthesis. Collectively, from the perspective of protein family evolution, these annotations elucidate the genetic basis underpinning the capabilities of MS11 in nutrient utilization, environmental tolerance, and ecological competition.

#### 3.3.4. CAZy Analysis

**Figure 6:**
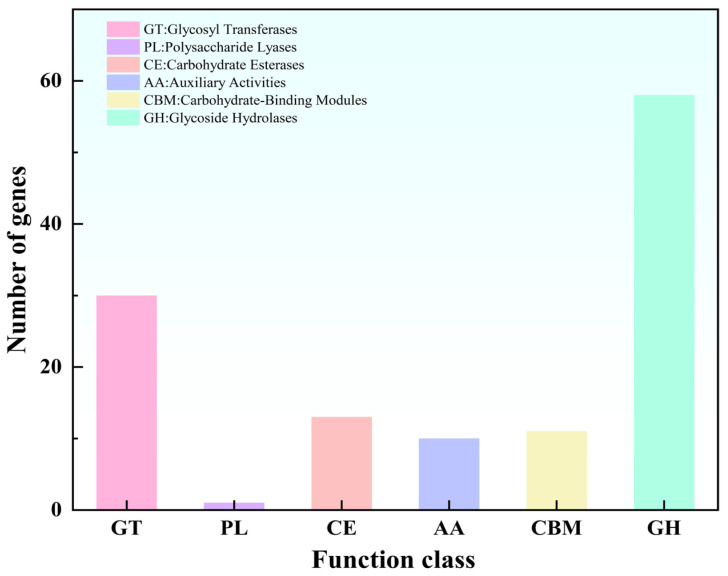
CAZy analysis.

Functional annotation against the CAZy database identified 123 genes encoding carbohydrate-active enzymes (CAZymes) in the genome of *L. plantarum* MS11 (Figure 6), indicative of substantial genetic diversity in carbohydrate metabolism. These genes were distributed across six major enzyme classes: glycoside hydrolases (GHs, 58 genes), glycosyl transferases (GTs, 30 genes), carbohydrate esterases (CEs, 13 genes), auxiliary redox enzymes (AAs, 10 genes), carbohydrate-binding modules (CBMs, 11 genes), and polysaccharide lyases (PLs, 1 gene). Among these, glycoside hydrolases (47.15%) and glycosyl transferases (24.39%) constituted the dominant components of the CAZyme repertoire, revealing molecular potential for the efficient degradation of complex carbohydrates and the synthesis of structurally diverse polysaccharides. This CAZyme profile underscores the metabolic versatility of MS11 in utilizing diverse carbon sources and synthesizing exopolysaccharides, while also providing key enzymatic insights into its mechanisms of functional adaptation within microecological niches.

#### 3.3.5. TCDB Analysis

**Figure 7:**
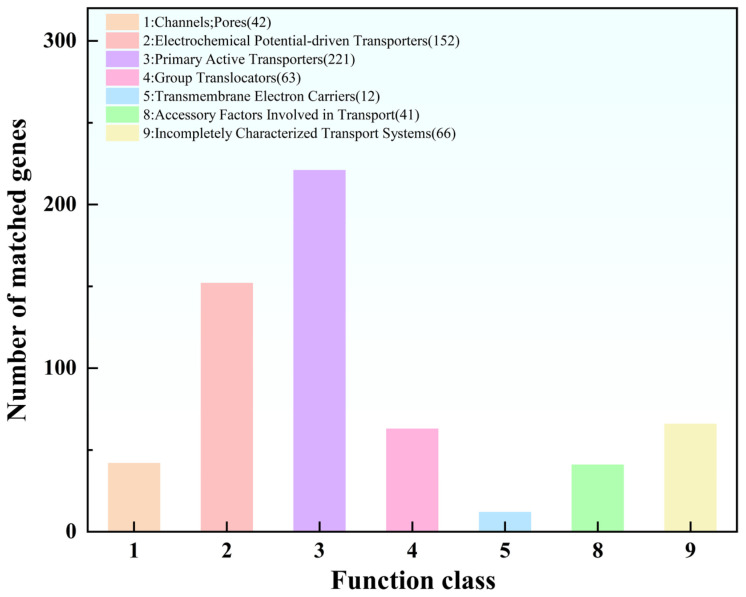
Chart of level 1 TCDB functional classification.

Annotation against the Transporter Classification Database (TCDB) identified 597 transporter-related genes in the MS11 genome, which were classified into seven major functional categories (Figure 7). Among these, electrochemical potential-driven transporters (152 genes) and primary active transporters (221 genes) constituted the core transmembrane transport system, underscoring efficient, energy-dependent substrate translocation capabilities. The genome also encoded a considerable number of pore-forming transporters (42 genes), group translocators (63 genes), transport accessory proteins (41 genes), and transmembrane electron carriers (12 genes). Together, these components form a multi-layered and synergistic substrate exchange network. Additionally, 66 genes were assigned to incompletely characterized or putative transport systems, suggesting the existence of unique or yet-to-be-defined transport mechanisms. Collectively, these results provide a systematic analysis of the transportoproteome, elucidating the molecular basis for the efficient metabolite uptake and environmental adaptation of MS11.

### 3.4. Biosafety Evaluation of L. plantarum MS11

Analysis against the Virulence Factors Database (VFDB) confirmed the absence of genes encoding known virulence factors—such as adhesins, invasins, toxins, or immune evasion factors—in the genome of *L. plantarum* MS11.

### 3.5. Genomic Information Mining of L. plantarum MS11

#### 3.5.1. Genetic Analysis of Folate Synthesis in *L. plantarum* MS11

**Figure 8:**
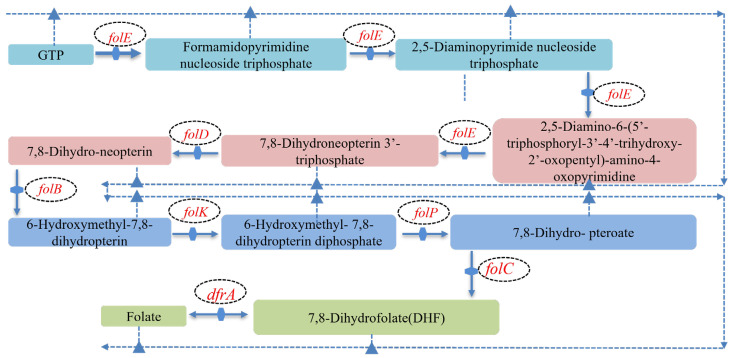
*L. plantarum* MS11 folate production gene pathway.

Integrated genomic and KEGG pathway analyses revealed that *L. plantarum* MS11 harbors a complete folate biosynthesis pathway (Figure 8). This pathway is initiated from guanosine triphosphate and proceeds through sequential catalysis by key enzymes—including *FolE*, *FolD*, *FolB*, *FolK*, *FolP*, *FolC*, and *DfrA*—ultimately yielding biologically active folate. The presence of this complete metabolic module provides a molecular basis for the potential development of MS11 as a nutritionally enhanced starter culture.

Genomic analysis confirmed that strain MS11 encodes 7 intact genes involved in folate biosynthesis (Table 3), indicating considerable potential for efficient folate production. This finding provides important genomic-level evidence for the role of lactic acid bacteria in host nutrient metabolism and regulation.

#### 3.5.2. Genetic Analysis of L-Lactic Acid Synthesis in *L. plantarum* MS11

**Figure 9:**
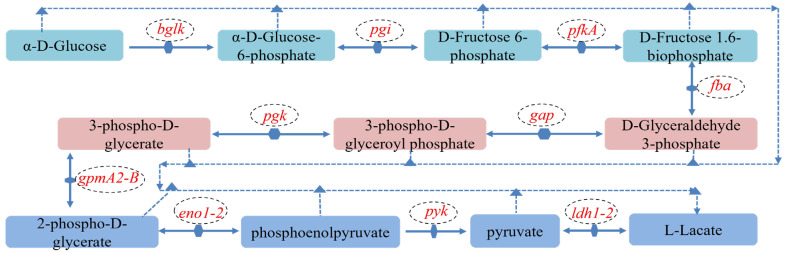
Genetic pathway for L-lactic acid production in MS11.

Integrated genomic and KEGG pathway analyses confirmed that *L. plantarum* MS11 harbors a complete glycolytic pathway. The strain efficiently utilizes monosaccharides such as glucose through the central glycolytic (Embden–Meyerhof–Parnas) pathway, converting them to pyruvate. Subsequently, pyruvate is converted predominantly to L-lactate by key enzymes such as lactate dehydrogenase, defining a typical homolactic fermentation phenotype (Figure 9). These characteristics underscore the considerable metabolic potential of MS11 as an efficient producer of L-lactate.

Genomic analysis identified 13 genes implicated in L-lactate synthesis (Table 4). Among these, the presence of two lactate dehydrogenase (LDH) genes, ldh1 and ldh2, suggests that lactate biosynthesis may be finely regulated. The presence of dual LDH gene copies may enable regulation of lactate stereospecificity via dosage effects or differential expression, potentially conferring an evolutionary advantage in metabolic flexibility and environmental adaptation.

#### 3.5.3. Genetic Analysis of Bacteriocins Synthesis in *L. plantarum* MS11

**Figure 10:**
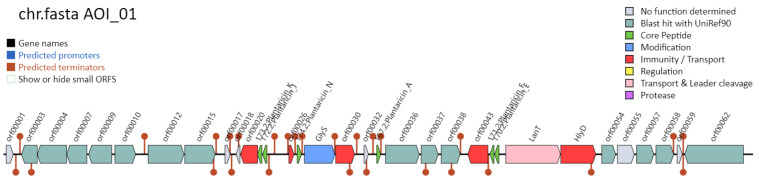
Prediction of bacteriocin biosynthetic gene clusters using BAGEL4.

In silico mining using the BAGEL4 platform (http://bagel4.molgenrug.nl/, 28 November 2025) identified a complete bacteriocin biosynthetic gene cluster spanning approximately 20.3 kb in the genome of *L. plantarum* MS11 as shown in Figure 10 [33]. This cluster comprises 14 putative functional genes responsible for bacteriocin synthesis, modification, transport, and regulation, suggesting a significant potential for bacteriocin production. This finding provides a genetic basis for the potential development of MS11 as a natural biopreservative.

Genomic annotation identified five genes encoding bacteriocin leader peptides in strain MS11: *plnA*, *plnJ*, *plnK*, *plnE*, and *plnF* (Table 5). Their predicted products contain the characteristic double-glycine cleavage site and a hydrophobic core, features consistent with Class II bacteriocin precursors. Genetic module analysis delineated a complete biosynthetic and regulatory network, in which *plnA*, *plnB*, and *plnC* mediate quorum-sensing regulation; *plnI* confers self-immunity; and *plnG*, *plnT*, *plnV*, and *plnW* are responsible for bacteriocin maturation and secretion. The presence of this complete plantaricin gene cluster confirms the bacteriocin-producing capability of MS11. This capability not only underscores the strain’s potential for ecological competition and pathogen inhibition but also provides a molecular framework for elucidating its antimicrobial mechanisms.

#### 3.5.4. Genetic Analysis of Exopolysaccharides Synthesis in *L. plantarum* MS11

**Figure 11:**
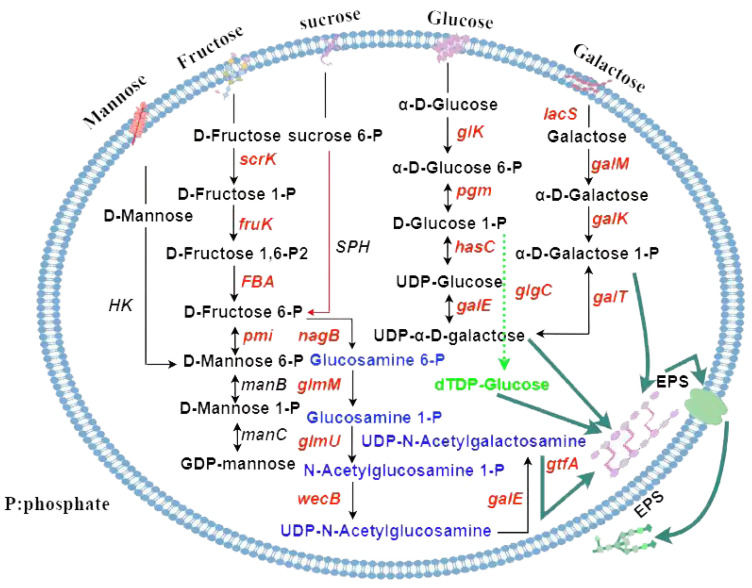
Nucleoside glycoside synthesis pathway.

The biosynthesis of exopolysaccharides (EPS) is a complex, multi-factorial process regulated by gene clusters, carbon sources, and pH [34,35]. The polymerization and secretion of EPS repeating units depend on dedicated transport systems. At the core of this process, amino sugar and nucleotide sugar metabolism serve as the primary source of glycosyl precursors and metabolic energy that fuel EPS assembly. KEGG pathway analysis predicted that strain MS11 harbors a versatile metabolic network for synthesizing nucleotide sugars from multiple carbon sources, including mannose, fructose, sucrose, glucose, and galactose (Figure 11). Among these, mannose, fructose, and sucrose are initially phosphorylated via the phosphoenolpyruvate-dependent phosphotransferase system (PEP-PTS). Through enzymatic reactions involving genes such as *scrK* and *fruK*, these sugars are channeled into central metabolic pools of fructose-6-phosphate and glucose-6-phosphate. These intermediates are subsequently converted into key nucleotide sugar precursors, including UDP-N-acetylgalactosamine, UDP-glucose, and dTDP-glucose. This integrated, multi-pathway metabolic capability underscores the adaptability of MS11 in sustaining EPS synthesis across diverse carbon sources.

Genomic annotation identified 17 genes implicated in exopolysaccharide (EPS) biosynthesis in strain MS11 (Table 6), a pathway known to require coordinated regulation of multiple genes and enzymes [36]. Among these, *gtfA* was annotated as a key enzyme gene crucial for EPS assembly. This multi-gene ensemble forms a complex biosynthetic network, reflecting a sophisticated genetic regulatory basis and metabolic plasticity underlying EPS production in this strain.

### 3.6. Analytical Validation of Metabolites in L. plantarum MS11

#### 3.6.1. Validation of Folate Production by *L. plantarum* MS11

**Figure 12:**
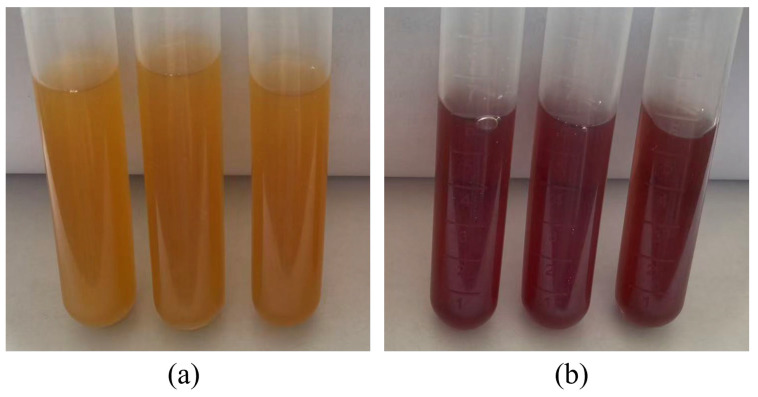
Chemotic diagram of Folate Biosynthesis in *L. plantarum* MS11. (**a**) The bacterial solution was added to the bromocresol purple indicator; (**b**) No bacterial solution was added to the bromocresol purple indicator.

Folate biosynthesis was assessed qualitatively using bromocresol purple in folate assay culture medium (FACM), as outlined in Figure 12. Triplicate tubes inoculated with an MS11 cell suspension (Figure 12a) were compared against uninoculated control tubes (Figure 12b). Following 24 h of incubation (37 °C), a distinct color change from yellow to purple was observed in the inoculated tubes concomitant with bacterial growth, confirming folate production and validating the experimental system for subsequent quantitative analysis.

**Figure 13:**
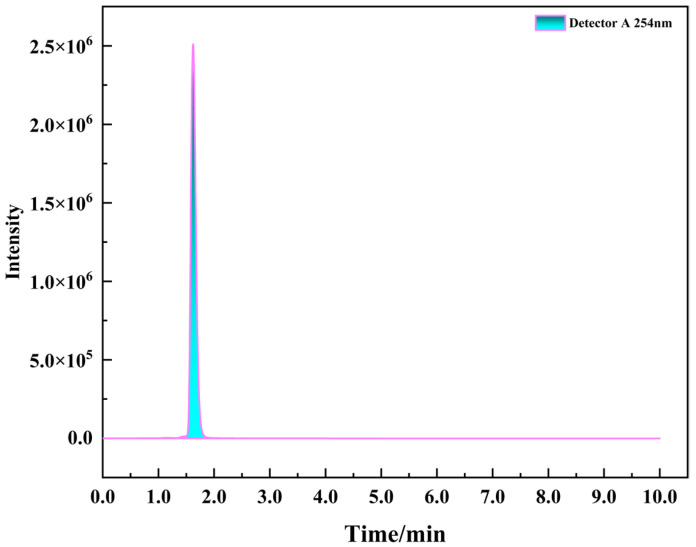
Chromatogram of the folate standard obtained by HPLC.

The chromatogram of a folate standard solution (0.25 μg/mL) obtained under the specified chromatographic conditions is presented in Figure 13. Folate eluted at a retention time of 1.62 min, displaying a symmetric peak shape and baseline separation from adjacent peaks. These observations confirm the effectiveness and specificity of the chromatographic method for folate quantification.

**Figure 14:**
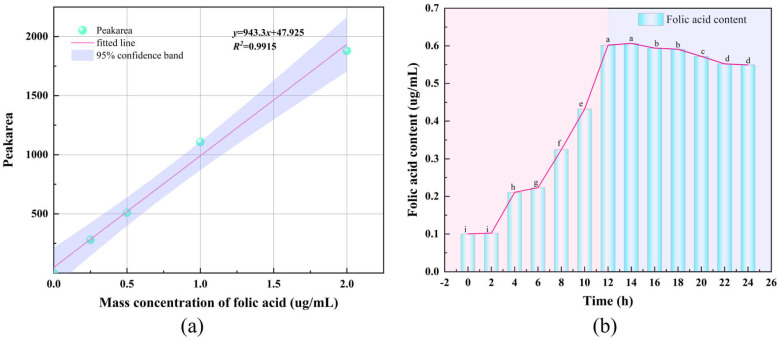
Standard curve for Folate and its production profile. Different lowercase letters within the same column indicate significant differences at *p* < 0.05. (**a**) Folic acid standard curve; (**b**) The content of folic acid produced by MS11 within 24 h.

A quantitative calibration curve was established by HPLC analysis of folate standard solutions at five concentrations (Figure 14a). The calibration curve was defined by the equation y = 943.3x + 47.925, with a coefficient of determination (R^2^) of 0.9915. Quantitative analysis showed that folate production by MS11 increased concomitantly with growth, reaching a maximum concentration of 0.6043 μg/mL at 12 h (Figure 14b). At this time point, the viable cell count reached 2.5 × 10^7^ CFU/mL, as determined by plate counting.

**Figure 15:**
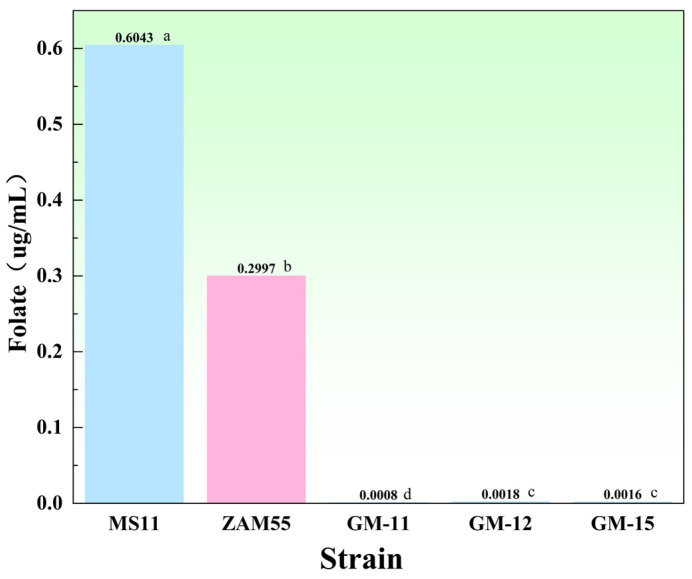
Comparison of folate content produced by different strains. Different lowercase letters within the same column indicate significant differences at *p* < 0.05.

The folate synthesis capabilities of five *L. plantarum* strains—MS11, ZAM55, GM-11, GM-12, and GM-15—were quantitatively compared. Significant inter-strain differences in folate production were observed (Figure 15). Strain MS11 demonstrated the highest production level. This was followed by strain ZAM55, which produced 0.2997 μg/mL [37], approximately 50% of the yield achieved by MS11. In contrast, strains GM-11, GM-12, and GM-15 showed markedly lower yields (0.0008, 0.0018, and 0.0016 μg/mL, respectively) [38]. Consequently, strain MS11 stands out as a highly promising candidate for microbial folate production.

#### 3.6.2. Validation of L-Lactic Acid Production by *L. plantarum* MS11

**Figure 16:**
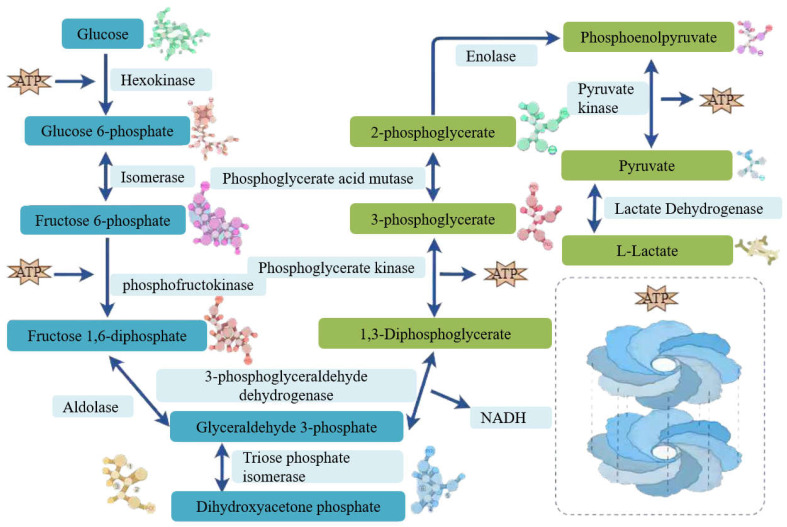
Schematic diagram of the acid production mechanism.

A core metabolic characteristic of *L. plantarum* is the production of organic acids, primarily lactic acid, via fermentation [39]. This process is primarily driven by the Embden–Meyerhof–Parnas (EMP) glycolytic pathway. Monosaccharides are catabolized to pyruvate, which is subsequently converted to lactate, resulting in an organic acid profile dominated by lactic acid ([40]; Figure 16). Through this conserved glycolysis–fermentation pathway, *L. plantarum* maximizes ATP yield to meet its basal energy demands while maintaining cellular redox balance. The lactate dehydrogenase (LDH)-catalyzed regeneration of NAD^+^ is crucial for sustaining glycolytic flux. From an ecological perspective, lactate accumulation lowers the extracellular pH, inhibiting the growth of competitors and thereby conferring a competitive advantage [5].

**Figure 17:**
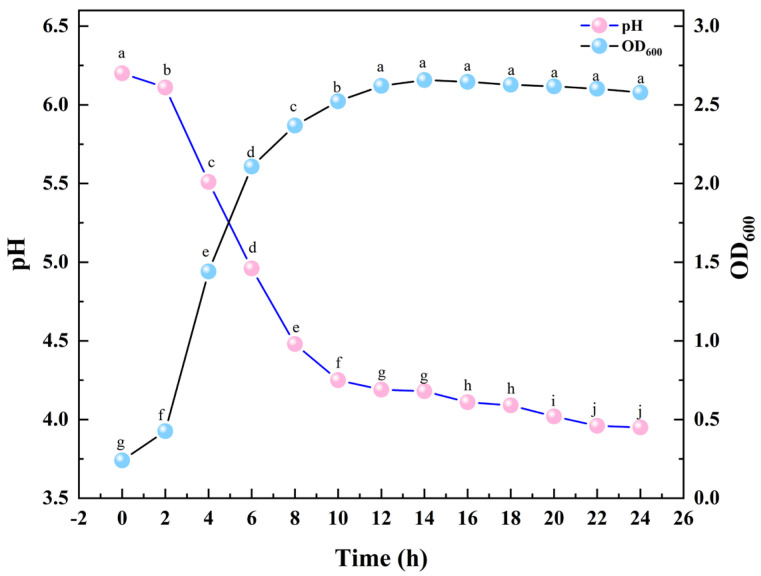
Growth and acid production curves of *L. plantarum* MS11. Different lowercase letters within the same column indicate significant differences at *p* < 0.05.

The growth curve and medium acidification (pH) profile of MS11 are shown in Figure 17. The growth curve indicates that MS11 entered the exponential phase after a 2 h lag period, exhibiting rapid proliferation. The late-exponential phase was reached by 10 h, after which the growth rate began to decline. The culture entered the stationary phase at approximately 12 h, during which the cell density stabilized. Concurrently, the pH of the culture medium decreased continuously throughout the growth phase. The pH stabilized and ceased to decline after approximately 16 h, coinciding with the entry into the late stationary phase. These results demonstrate that MS11 exhibits peak metabolic activity during the exponential growth phase.

**Figure 18:**
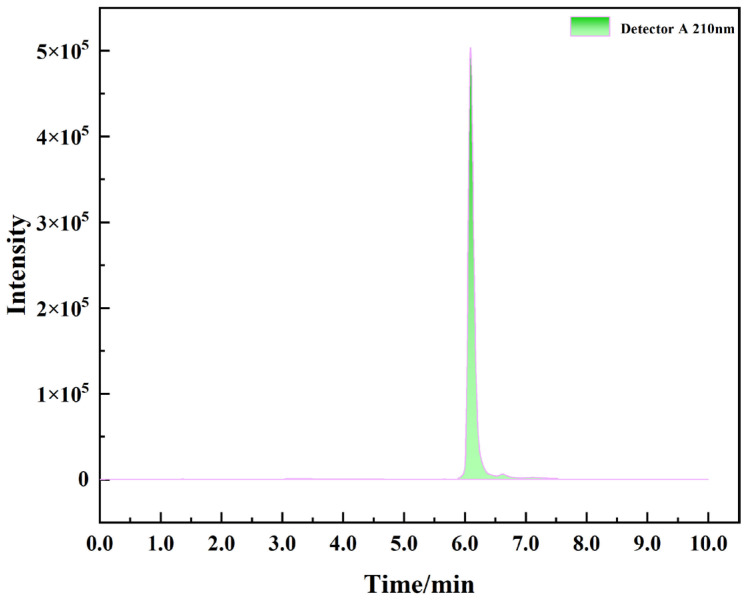
Chromatogram of the L-Lactic acid standard obtained by HPLC.

The chromatogram for an L-lactate standard (1.0 mg/mL) obtained under the specified conditions is presented in Figure 18. L-lactate eluted at a retention time of 6.12 min, displaying a symmetrical, sharp peak with baseline separation from adjacent components. This confirms the effectiveness and specificity of the chromatographic method for the quantification of L-lactate.

**Figure 19:**
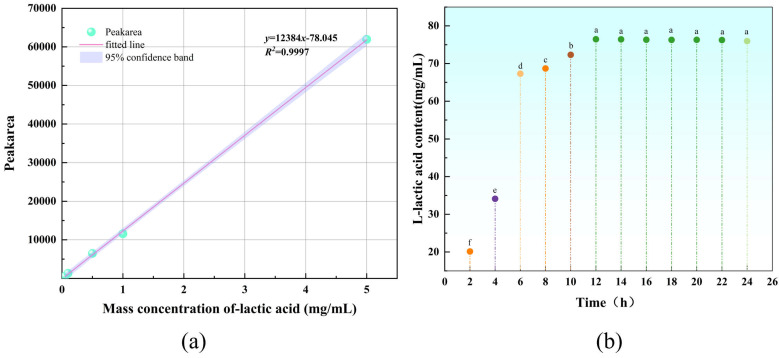
Standard curve for L-Lactic acid and its production profile. Different lowercase letters within the same column indicate significant differences at *p* < 0.05. (**a**) Lactic acid standard curve; (**b**) The lactic acid content produced by MSII within 24 h; The different colored dots in (**b**) represent the lactate content.

A quantitative calibration curve for L-lactate was established by HPLC analysis of standard solutions at five concentrations (Figure 19a). The calibration curve was defined by the equation y = 12384x − 78.045 (R^2^ = 0.9997). Analysis of fermentation samples showed that L-lactate production by MS11 increased concomitantly with growth, reaching a maximum concentration of 76.24 mg/mL at 12 h (Figure 19b). At this time point, the viable cell count reached 1.3 × 10^7^ CFU/mL, as determined by plate counting.

#### 3.6.3. Validation of Bacteriocins Production by *L. plantarum* MS11

**Figure 20:**
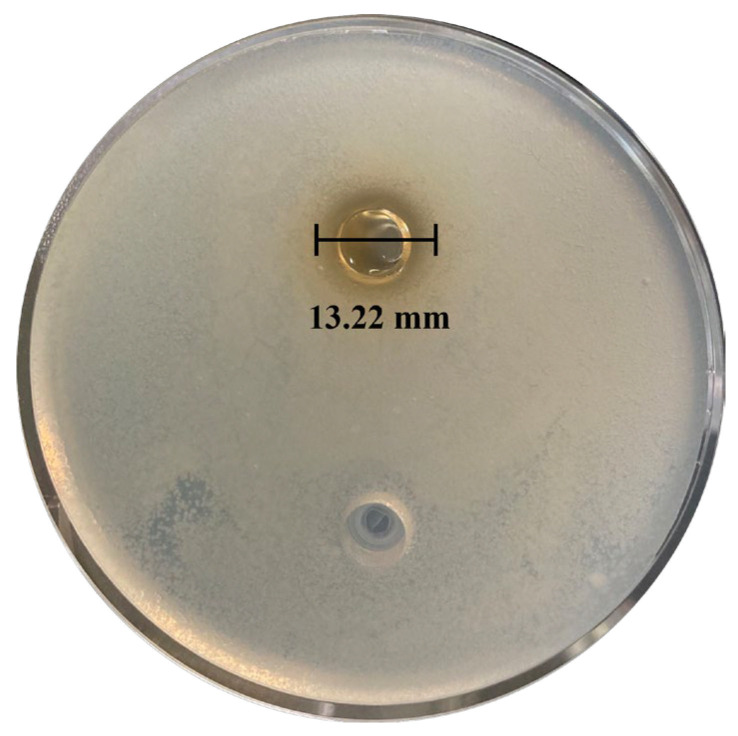
*L. plantarum* MS11 Inhibition of bacteriocin production on *E. coli*.

From the fermentation broth, 1.46 g of crude bacteriocin extract was obtained. The antibacterial activity of the crude extract was evaluated against Escherichia coli using the double agar diffusion assay, which yielded a clear inhibition zone of 13.22 mm in diameter (Figure 20). Furthermore, the extract exhibited significant inhibitory activity against a range of food spoilage Gram-positive bacteria. This broad-spectrum activity is consistent with the genomic prediction of a diverse repertoire of bacteriocin biosynthetic genes in MS11.

#### 3.6.4. Validation of Exopolysaccharides Production by *L. plantarum* MS11

**Figure 21:**
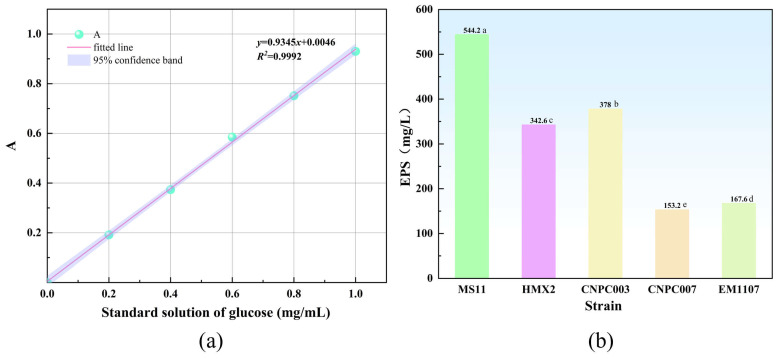
Glucose standard curve and comparison of EPS content produced by different strains. Different lowercase letters within the same column indicate significant differences at *p* < 0.05. (**a**) Glucose standard curve; (**b**) Comparison of EPS production by different strains.

A standard calibration curve for glucose quantification was constructed via the phenol-sulfuric acid method, yielding a linear regression of y = 0.9345x + 0.0046 with a high coefficient of determination (R^2^ = 0.9992) across a five-point concentration range (Figure 21a). Building upon the genomic potential indicated by KEGG annotation, the EPS production by MS11 was experimentally quantified, revealing a substantial yield of 544.2 mg/L.

EPS production was quantitatively compared across five *L. plantarum* strains: MS11, HMX2, CNPC003, CNPC007, and EM1107. The results revealed substantial inter-strain variation in EPS yield (Figure 21b). MS11 demonstrated the highest production capacity. Strain CNPC003 ranked second with a yield of 378 mg/L, approximately 43.97% lower than that of MS11. The yields of CNPC007 and EM1107 were 153.2 mg/L and 167.6 mg/L, respectively [41], while HMX2 produced 342.6 mg/L [42]. These findings confirm MS11 as a superior and highly promising EPS-producing strain.

## 4. Discussion

### 4.1. Genotype-Phenotype Adaptability Driven by High-Altitude Niche

The *L. plantarum* MS11 strain characterized in this study harbors the complete genetic pathways and exhibits an efficient phenotype for the concurrent production of lactic acid, exopolysaccharides (EPS), bacteriocins, and folate. This multifunctional profile can likely be attributed to adaptive evolution within its unique ecological niche: traditionally fermented yak milk from the high-altitude Gannan region. The persistent low-temperature environment may have selected for strains with enhanced capabilities in lactic acid production (to rapidly lower pH) and EPS synthesis (for cryoprotection and biofilm formation). Concurrently, the nutrient-limited and non-sterile conditions of the fermentation process would favor the retention of folate biosynthesis (as a nutritional determinant) and bacteriocin production (for microbial competition). This aligns with the established principle that environmental niches drive significant genetic diversity and metabolic specialization in microbial populations [43,44]. Isolates from high-altitude or other extreme environments often possess distinctive genomic adaptations. To thrive in the competitive and resource-limited habitat of fermented yak milk, MS11 likely evolved a coordinated genetic repertoire enabling the synthesis of this suite of complementary functional metabolites, a trait strongly favored by natural selection. Supporting the concept of niche-specific genetic shaping, a comparative genomic analysis reveals a difference in carbohydrate metabolic potential: *L. plantarum* SK4791, isolated from fermented leeks, encodes 47 glycoside hydrolase genes [45], whereas MS11 possesses 58 such genes. This comparison underscores how the environmental niche substantially influences the adaptive genetic repertoire of bacterial strains.

### 4.2. Convergence and Efficient Expression of Multifunctional Metabolic Phenotypes

The high-yield production of L-lactic acid by *L. plantarum* MS11 serves a dual purpose: it is a central fermentation product and, by rapidly acidifying the environment, creates a synergistic antimicrobial barrier when combined with bacteriocins. Bacteriocin production in MS11 is likely regulated by a quorum-sensing mechanism, synchronizing gene expression with high cell density to deploy a coordinated antimicrobial defense [46,47]. This strategy is consistent with reports that fermentation enhances the antimicrobial activity of LAB [48,49], a synergy between acidification and bioactive compounds that is central to food biopreservation [50]. These combined traits highlight the potential of MS11 in applications ranging from fermented food production to industrial lactic acid fermentation [51,52]. Simultaneously, EPS synthesis is crucial for protective biofilm formation and survival under stressful conditions, such as the low temperatures of its high-altitude niche. Genomic analysis confirms that MS11 possesses the necessary glycosyltransferase and polymerase genes for EPS assembly [53]. MS11 produced 57.41% more EPS than *L. plantarum* MC5 [32], confirming its status as a high-yield EPS-producing strain. As EPS synthesis is energy-intensive, future fermentation optimization must critically balance biomass growth, acid production, and polysaccharide yield [21]. Furthermore, efficient folate biosynthesis likely enhances the nutritional profile of the fermented product and may provide a competitive metabolic advantage within the microbial community of yak milk. For instance, *L. plantarum* A3 produces 0.6421 ± 0.062 μg/mL of folate [54]. MS11, with a yield of 0.6043 μg/mL, is a comparably high producer. The unique value of MS11 lies in its ability to concurrently produce high levels of multiple functional metabolites. This trait is highly strain-dependent. For example, among folate-producing Streptococcus thermophilus strains, only two (K73 and K47) achieved the highest yields, highlighting significant inter-strain variability [29]. Using MS11 as a starter culture to develop folate-biofortified foods presents safety and bioavailability advantages over chemical fortification [34,55].

### 4.3. Metabolic Interactions and Regulatory Basis Underlying Multi-Product Synthesis

The efficient and concurrent production of multiple metabolites in MS11 implies a sophisticated regulatory system for partitioning carbon flux and orchestrating global gene expression. Notably, MS11 achieves exceptionally high titers of lactic acid (76.24 mg/mL) while concurrently sustaining substantial EPS production (544.2 mg/L)—a metabolically costly process. This suggests a finely tuned mechanism that balances glycolytic flux with the diversion of precursors for EPS biosynthesis, indicating a cooperative rather than purely competitive metabolic relationship. Rapid acidification via glycolysis serves a dual role: it generates ATP and lowers the extracellular pH, creating a broad-spectrum inhibitory environment for competitors. Building upon this foundational defense, the synthesis of specific bacteriocins provides a targeted antimicrobial capacity, further alleviating niche competition. Concurrently, secreted EPS facilitates the formation of a protective biofilm or capsule, maintaining membrane integrity and hydration to counteract environmental stresses such as low temperature, desiccation, and oxidative stress [35]. Meanwhile, de novo folate biosynthesis supports essential cellular processes like nucleic acid synthesis, promoting growth under nutrient limitation, and may also serve as a public good to modulate microbial community dynamics [56].Thus, these metabolites do not function in isolation but constitute an integrated, multi-layered adaptive network. This integrated network represents a strategy of metabolic synergy that collectively enhances ecological fitness and resilience in challenging environments [57].

### 4.4. Study Limitations and Future Perspectives

This study provides a preliminary elucidation of the phenotypic and genetic basis underlying multi-product synthesis. However, how these metabolic pathways are coordinately regulated at the transcriptional and translational levels remains to be elucidated. Future research may involve conducting transcriptomic analysis under simulated stress conditions of yak milk fermentation to identify key regulatory genes, as well as constructing deletion mutants of key genes involved in folate or EPS biosynthesis to quantify their specific contributions to competitive fitness within the ecological niche using a co-culture model.

As a core strain in starter cultures, *L. plantarum* often coexists with other lactic acid bacteria in practical applications. Its growth-promoting or inhibitory effects on other lactic acid bacteria are important indicators for evaluating its application potential. Future studies should therefore prioritize co-culture experiments to further refine the application evaluation of this strain.

## 5. Conclusions

In this study, by employing a strategy that integrates whole-genome sequencing with experimental validation, we systematically elucidated the metabolic genetic basis and functional characteristics of *L. plantarum* MS11—a strain isolated from traditionally fermented yak milk in a high-altitude region that exhibits high-yield production of folate, L-lactic acid, bacteriocins, and exopolysaccharides (EPS)—and further revealed its application potential as a multifunctional probiotic. The main conclusions are as follows:

(1) Genomic characterization showed that the *L. plantarum* MS11 genome consists of a single circular chromosome and three plasmids, totaling 3,318,231 bp with a GC content of 44.48%, and encodes 3155 predicted proteins. Crucially, it harbors complete biosynthetic gene clusters for folate, L-lactic acid, bacteriocin, and exopolysaccharides (EPS), which together form the genetic foundation for its multifunctional metabolic phenotype.
(2) Experimental validation confirmed the efficient biosynthesis of folate (0.6043 μg/mL) by MS11, along with the concomitant production of L-lactic acid (76.24 mg/mL), bacteriocin (1.46 g crude extract), and exopolysaccharides (544.2 mg/L). The notably high titers of folate and L-lactic acid, coupled with the substantial yield of EPS, provide a robust material basis for its development in applications such as nutritional fortification, acidification, and bio-thickening.
(3) *L. plantarum* MS11 is a multifunctional strain characterized by the efficient production of folate, high-titer L-lactic acid, substantial EPS yield, and pronounced antimicrobial activity. This synergistic metabolic profile underscores its considerable potential for development as a multifunctional starter culture, a natural bio-preservative, and a nutritionally fortified probiotic.

This study establishes a genomic and phenotypic foundation for mining probiotic resources from unique ecological niches and identifies *L. plantarum* MS11 as a promising candidate for developing high-value fermented foods and functional preparations.

## Figures and Tables

**Table 1:** General features of the *L. plantarum* MS11 genome.

Type	Seq Length (bp)	Num of ORF	GC Content (%)	ORF/Genome (Conding Percentage) (%)
Chromosome	3,318,231	3155	44.48	83.59
Plasmid1	76,909	79	37.94	77.15
Plasmid2	13,687	17	35.73	55.72
Plasmid3	3210	3	38.72	74.58

**Table 2:** Summary of functional annotation for genes from *L. plantarum* MS11.

Name	Database						
	NR	eggNOG	KEGG	GO	Swiss-Prot	Pfam	TCDB
*L. plantarum* MS11	3147	2655	1424	2220	2110	2389	597

**Table 3:** Folate-associated genes in *L. plantarum* MS11.

Locus Tag	Gene	Amino Length	KEGG_ID
chr_2858	*folE*	150	K01495
chr_2017	*folC*	444	K11754
chr_2855	*folP*	382	K00796
chr_1596	*dfrA*	163	K00287
chr_2859	*folK*	161	K00950
chr_2860	*folB*	122	K01633
chr_1364	*folD*	286	K01491

**Table 4:** Acid-associated genes in *L. plantarum* MS11.

Locus Tag	Gene	Function	KEGG ID
chr_390	*bglk*	Convert glucose to glucose-6-phosphate	K18673
chr_2105	*pgi*	Convert glucose-6-phosphate to fructose-6-phosphate	K01810
chr_1620	*pfkA*	Convert fructose-6-phosphate to fructose-1, 6-diphosphate	K00850
chr_301	*fba*	Convert fructose-1, 6-diphosphate to glyceraldehyde-3-phosphate and dihydroxyacetone phosphate (DHAP)	K01624
chr_686	*gap*	Convert glyceraldehyde-3-phosphate to 1, 3-diphosphoglyceric acid	K00134
chr_687	*pgk*	Convert 1, 3-diphosphoglyceric acid to 3-phosphoglyceric acid	K00927
chr_2691	*gpmA2*	Convert 3-phosphoglyceric acid to 2-phosphoglyceric acid	K01834
chr_845	*gpmB*	Convert 3-phosphoglyceric acid to 2-phosphoglyceric acid	K02226
chr_689	*eno1*	Convert 2-phosphoglycerate to phosphoenolpyruvate (PEP)	K01689
chr_1638	*eno2*	Convert 2-phosphoglycerate to phosphoenolpyruvate (PEP)	K01689
chr_1619	*pyk*	Convert PEP to pyruvate	K00873
chr_462	*ldh1*	lactate dehydrogenase, partial	K00016
chr_942	*ldh2*	lactate dehydrogenase, partial	K00024

**Table 5:** Bacteriocin-associated genes in *L. plantarum* MS11.

Locus Tag	Gene	Amino Length	Gene Function
chr_361	*PlnK*	57	Dual-peptide bacteriocin plantaricin
chr_362	*PlnJ*	53	Two-peptide bacteriocin plantaricin
chr_365	*PlnO*	399	Glycosyl transferase group 2 family
chr_367	*PlnQ*	62	Putative protein with unknown function
chr_368	*PlnA*	48	Bacteriocin plantaricin-A, Induction pheromone
chr_369	*PlnB*	442	Histidine protein kinase
chr_370	*PlnC*	247	Response regulator
chr_372	*PlnI*	257	Bacteriocin immunity protein
chr_373	*PlnF*	52	Dual-peptide bacteriocin plantaricin
chr_374	*PlnE*	56	Two-peptide bacteriocin plantaricin
chr_375	*PlnG*	716	Bacteriocin ABC-transporter
chr_377	*PlnT*	181	Integral membrane protein, membrane-bound protease CAAX family
chr_379	*PlnV*	226	Putative protein harboring a CAAX protease motif
chr_380	*PlnW*	228	Putative protein containing a protease CAAX signature

**Table 6:** EPS-associated genes in *L. plantarum* MS11.

Locus Tag	Gene	Amino Length	KEGG_ID
chr_1085	*gtfA*	498	K00712
chr_415	*glmU*	460	K04042
chr_714	*glmM*	451	K03431
chr_488	*wecB*	245	K05946
chr_207	*nagB*	237	K02564
chr_1822	*fruK*	305	K00882
chr_301	*FBA*	287	K01624
chr_2995	*lacS*	652	K11104
chr_19	*glgC*	375	K00975
chr_3111	*scrK*	287	K00847
chr_2076	*pmi*	321	K01809
chr_719	*galM*	339	K01785
chr_2989	*galT*	487	K00965
chr_2990	*galE*	334	K01784
chr_2991	*galK*	387	K00849
chr_654	*hasC*	306	K00963
chr_666	*pgm*	575	K01835

## Data Availability

The original contributions presented in this study are included in the article. Further inquiries can be directed to the corresponding author.
